# The role of negative incisional pressure in the prevention of surgical site complications in patients with median incisional hernia

**DOI:** 10.1186/s40001-025-02724-y

**Published:** 2025-06-07

**Authors:** Francesco Marena, Tito Brambullo, Lorenzo Montrasio, Nicola Baldan, Francesca Mancini, Vincenzo Vindigni, Franco Bassetto

**Affiliations:** 1 Clinic of Plastic Surgery, Department of Neurosciences, University of Padua, Address Via Giustiniani 2, Padua, Veneto Italy; 2 1 st Surgical Clinic, Department of Surgery, Oncology and Gastroenterology, University of Padua, Padua, Italy

**Keywords:** Incisional hernia, Single-use negative pressure, Surgical site occurrences, Abdominal wall reconstruction

## Abstract

**Background:**

A single-center retrospective study was conducted to investigate whether the prophylactic application of a single-use negative pressure wound therapy (sNPWT) dressing on closed surgical incisions following incisional hernia (IH) repair of the abdominal wall with meshes reduces the risk of surgical site occurrence (SSO) and the necessity for surgical reoperation.

**Methods:**

A retrospective study was conducted on 55 patients with incisional hernias classified as W2 (> 4–10 cm) or W3 (> 10 cm) according to the European Hernia Society classification, treated between 2013 and 2023. All patients underwent open surgical repair with mesh and were assigned to either a conventional flat dressing group (*n* = 34) or an sNPWT group using PICO 7 (*n* = 21). Weekly follow-ups were performed, and outcomes were statistically analyzed to compare the incidence of SSOs and reoperations between the two groups.

**Results:**

At 30 days postoperatively, the control group showed a higher incidence of SSOs (32.35%, 11 cases) compared to the PICO 7 group (19.05%, 4 cases, *P* = 0.28). The need for surgical reintervention was also higher in the control group (17.65%, 6 cases) versus the PICO 7 group (10.53%, 2 cases, *P* = 0.41). Regardless of dressing type, elevated BMI (*P* = 0.02), advanced age, and diabetes were identified as key risk factors for SSOs.

**Conclusions:**

sNPWT with PICO 7 may reduce SSOs and reoperations in open incisional hernia repair, particularly in high-risk patients with elevated BMI. Although statistical significance was not achieved, sNPWT appears to be a valuable adjunct in postoperative management. Further research is necessary to confirm its efficacy and determine the ideal patient population.

## Background

Incisional hernia results from a weakness of the abdominal wall at the site of prior incision and closure after surgery. It has been calculated to occur in approximately 10–19% of patients undergoing laparotomy depending on the fascial closure technique adopted [1], representing the most common complication that requires reoperation [2]. Surgical technique is an important risk factor for the development of incisional hernia, in particular midline incisions have the highest incidence (3–20%) if compared to paramedian, transverse [3–5] and laparoscopic incisions. The recurrence rate is between 23 and 50% [6] and it has been estimated that a reduction in incidence of incisional hernia by 5% resulted in a cost saving of 4 million per year in France.

A study by Poulose et al. [7] estimated the average expense for each inpatient surgical procedure in the United States to be USD$15,899 in 2006, resulting in a yearly total of approximately $3.2 billion. However, Bower and Roth [8] suggested that this figure likely underestimates the full costs, as it excludes physician fees and societal expenses such as lost work time.

In the process of closing a laparotomy incision, the linea alba is reunited, and the rectus muscles are restored to their midline position. The repair's effectiveness depends on suture fixation until scar tissue develops enough strength to match or surpass the suture material. The primary cause of ventral incisional hernia formation is the lateral displacement of the rectus muscle, which results in a deficit of tension, causing the protrusion of omental fat or intestinal loop in the subcutaneous plane [9]. Surgical mesh has become the preferred method for incisional hernia repair, addressing loss of domain and helping maintain optimal rectus muscle function at the midline. A multicenter randomized study in the New England Journal of Medicine demonstrated the efficacy of mesh. Luijendijk et al. [10] discovered that patients who underwent traditional suture repair had a recurrence rate nearly double that of those who received mesh repair. Furthermore, a recent meta-analysis in the Journal of the American Medical Association, Surgery, showed that suture repair patients experienced hernia recurrence rates almost three times higher than those who underwent mesh repair [11]. As a result, the Ventral Hernia Working Group now recommends using mesh to reinforce all ventral hernia repairs [12]. They also suggest centralizing and reapproximating the paired rectus muscles. In cases where the rectus muscles are significantly separated and cannot be easily brought together at the midline, component separation may be beneficial. This technique involves partially releasing the abdominal wall fascia connecting the oblique and rectus muscles [13].

The most frequent complication following surgical mesh use is hernia recurrence [9, 11, 12]. Essentially, recurrence occurs due to early mesh degradation, premature mesh removal (often necessary after infections), or mesh failure [13, 14]. Unlike primary abdominal wall hernias, incisional hernias have diverse dimensions and forms. Consequently, a single variable or measurement cannot adequately capture the size of the incisional hernia. To classify these hernias in a two-dimensional grid format, it is crucial to condense the “size of the hernia defect” into one quantitative or semi-quantitative measure. Chevrel addressed this challenge by selecting the hernia defect’s width as the primary classification parameter, asserting that width is the most critical size measurement for determining the difficulty of successful hernia repair [15].

In 2009, the European Hernia Society defined the current classification of ventral hernias [16], while in 2010, the Ventral Hernia Working Group introduced a grading system aimed at stratifying the risk of wound complications and recurrences in incisional hernias, termed surgical site occurrence (SSO). In 2024, Anwoju et al. [17] followed through with the European Hernia Society Classification of Ventral Hernias, which established more specific outcomes in specific groups through a comprehensive retrospective study.

This system is commonly employed to provide a decision-making framework for patients with incisional hernias. However, many researchers concur that its applicability is limited to open surgery, because the risk factors for SSO differ slightly in the context of Laparoscopic Ventral Hernia Surgery.

Single-use or incisional negative pressure wound therapy (sNPWT or iNPWT) works by applying controlled negative pressure to the wound surface, which can help to remove excess fluid, reduce edema, and promote perfusion, ultimately contributing to improvement. This therapy has been applied in other medical fields, including orthopedic surgery, cardiothoracic surgery, and plastic surgery, where wound management and the prevention of post-surgical complications are critical.

Studies indicate that NPWT may reduce the incidence of surgical site infections (SSI) by creating a closed environment that minimizes bacterial contamination and supports tissue regeneration [18].

Negative pressure dressings have been used in a number of different surgical setting with promising results [19–21].

The aim of this study is to assess the impact of using single-use negative pressure wound therapy (sNPWT) on preventing surgical site complications (SSOs) as the primary endpoint, and on reducing the need for reoperation as the secondary endpoint. When prosthetic materials are utilized, preventing complications becomes even more critical and cost-effective.

## Materials and methods

We conducted a retrospective study including 55 patients with primary midline incisional hernias classified as type W2 (4–10 cm) or W3 (greater than 10 cm) according to the European Hernia Society classification (Table 1). All patients underwent open surgical repair with prosthetic mesh placement. The procedures were standardized and performed by a specialized surgical team.

**Table 1:** Incisional hernia classification

Width (cm)	W1	W2	W3
range	< 4 cm	≥ 4–10 cm	≥ 10 cm

Classification of incisional hernia based on the width of the defect according to the European Hernia Society. Table created by the authors with citation of Muysoms et al. [27]

### Study design

This retrospective matched cohort analysis involved 55 patients who underwent complex incisional hernia repair (IHR) at our university hospital between January 2013 and May 2023. We included adult patients undergoing elective, open primary IHR with mesh, specifically for hernias classified as W2 or W3 per the European Hernia Society criteria. We selected patients with no history of wound infections, all of whom were treated in a clean-contaminated environment. Cases involving stomas or contaminated wounds, patients with active neoplasm or undergoing radiotherapy or chemotherapy were excluded. Patients with less than 30 days of postoperative follow-up were also excluded. Post-surgery, patients received either conventional flat dressings (control group; *n* = 34) or sNPWT (PICO 7) dressings (*n* = 21). PICO 7 (Smith & Nephew, London, UK) is a single-use negative pressure dressing applied for the first seven postoperative days, each of the two groups received patients who were randomly assigned to them.

The study received approval from our Institution Ethics Committee (CET-ACEV 532n/AO/24), in compliance with data protection regulations.

### Surgical technique

All patients received antibiotic prophylaxis including Cephalosporine 1# generation as first choice and Clindamicin in case of known allergy. The type of repair was chosen based on hernia characteristics and surgeon expertise, with retro-rectus repair preferred where possible. Component separation was routinely performed in cases where tension-free fascial approximation was unachievable, with transversus abdominis release (TAR) as the preferred method if necessary. Mesh reinforcement was applied in all cases with extraperitoneal placement (Table 2).

**Table 2:** Characteristics of mesh and plane of location in the study groups

Mesh and plane	Control group (*n* = 34)	PICO 7 group (*n* = 21)
Syntetic non-absorbable (Versatex™ Medtronic; Prolene® Ethicon J&J MedTech)	31 (91.1%)	14 (66.6%)
Syntetic partially absorbable (Proceed® Ethicon J&J MedTech)	1 (2.9%)	1 (4.7%)
Syntetic full absorbable (Bio-A® Gore medical)	1 (2.9%)	2 (9.5%)
Acellular dermal matrix (Egis® DecoMed srl; Permacol™ Medtronic)	0	4 (19%)
Double syntetic mesh* (full absorbable + non-absorbable)	1 (2.9%)	9 (42.8%)
Onlay	0	1 (4.7%)
Retrorectus	11 (32.3%)	8 (38%)
Retromuscular	22 (64.%)	11 (52.3%)
Intraperitoneal	2 (5.8%)	9 (42.8%)
anterior separation component	1 (2.9%)	0
posterior separation component	22 (64.7%)	12 (57.1%)

Values are reported as mean ± standard deviation or absolute and percentage frequency

^*^ Full absorbable mesh was used to reconstruct the peritoneal layer and considered intraperitoneal, while the non-absorbable was located in the retromuscular plane after transversus abdominis muscle release and considered retromuscular

### Intervention

The sNPWT dressing (PICO7®) was applied to the surgical site by the operating surgeon, with self-adherent tape used to ensure an airtight seal around the dressing. Negative pressure was activated using the attached vacuum unit (Smith & Nephew® Srl, Italy). The control group received standard flat dressing. Postoperative antibiotics were administered routinely for 1 week.

### Outcomes and covariates

Patient data were stored in a dedicated database, with ethics committee approval and adherence to data protection laws. Patients with active infection, a history of wound infection, active oncological disease, lack of weekly follow-up, or lack of consent for data processing were excluded. Collected data included sex, age, body mass index, risk factors (diabetes, smoking, anemia, immunosuppression), and postoperative events (including wound dehiscence and reinterventions). Operative details, including type of component separation, mesh use, material, and positioning, were recorded.

This study focused on patients with a low risk of complications and no wound infection history (Grade 1) all treated in a clean-contaminated setting (Grade 3, Category A). We selected specific risk factors, among the ones outlined in Grade 2 and others presented in studies on surgical site healing [22] (see Table 3), to better stratify patients and compare the two groups.

**Table 3:** Risk factors for surgical site occurrences

Risk factor	range
Body Mass Index	≥ 30
Age	≥ 65 years
Diabetes	≥ 126 mg/dL (7.0 mmol/L)
Smoking	active smoking or cessation of less than 6 months
Anemia	≤ 10 g/dL
Immunosuppression	white blood cell count ≤ 4.5 × 109/L

Table made by the authors with citation of Berger et al., Kanters et al. and Marchica et al.[22–24]

### Follow-up and endpoint assessment

All patients received weekly follow-up consultations until complete healing. During each visit, a specialized team conducted physical examinations to assess the potential onset of surgical site occurrences (SSOs), including dehiscence, infection, hematoma, and seroma (primary endpoint). All complications were recorded and managed conservatively or, when necessary, with surgical reintervention (secondary endpoint).

### Statistical analysis

The statistical analysis included both descriptive and inferential methods. Data analysis was performed using Microsoft Excel and Statistics Kingdom software. Quantitative variables were reported as means and standard deviations, while qualitative variables were expressed as absolute and percentage frequencies. The Shapiro–Wilk test was used to assess the normality of distributions. The Student’s *t* test was applied to normally distributed data, while the Mann–Whitney test was used for non-normally distributed data. Categorical variables were compared using the chi-square test. An alpha level of 0.10 was set, and *p* values below 0.05 were considered statistically significant.

## Results

From the assessment of the primary endpoint (surgical site occurrence), were 11 cases in the control group, representing 32.35% of the cases. In contrast, the group treated with the PICO7® dressing experienced four cases of dehiscence, corresponding to 19.05% (*P* = 0.28). Similarly, for the secondary endpoint (need for surgical reoperation due to complications), the control group had six reoperations, representing 17.65% of cases. In contrast, the PICO7® group had 2 reoperations, corresponding to 10.53%. Despite the apparent difference, this parameter also did not reach statistical significance (*P* = 0.41) (Table 4).

**Table 4:** Endpoints of the study

Outcome	Control group (*n* = 34)	PICO 7 group (*n* = 21)	*p* value
Primary endpoint—SSO	11 (32.35%)	4 (19.05%)	0.28
dehiscence	4 (11.76%)	1 (4.76%)	0.38
infection	3 (8.82%)	2 (9.52%)	0.93
hematoma	3 (8.82%)	1 (4.76%)	0.57
seroma	1 (2.94%)	0	0.43
Secondary endpoint—need for surgical reoperation	6 (17.65%)	2 (10.53%)	0.41

Values are reported as absolute and percentage frequency. *p* value was calculated as indicated in “Materials and methods” section

Since BMI was the only significant risk factor related to surgical site occurrence, we assessed the outcomes for obese patients to evaluate the risk of SSOs in the control group compared to the intervention group treated with the Pico system. In the control group, consisting of 12 obese patients, 7 experienced SSOs, resulting in an overall risk of 58.3%. Conversely, in the intervention group, which included 5 obese patients treated with the Pico system, only 2 developed SSOs, corresponding to a related risk of 40% (*P* = 0.40).

The analysis identified the anthropometric data and risk factors that were most likely linked to surgical site complications, particularly the risk of dehiscence and infections (Table 5).

**Table 5:** Anthropometric data and risk factors associated with SSO

Risk factor	Presence of SSO	Absence of SSO	*p* value
Body mass index (kg/m^2^)	31.9 ± 5.3	27.1 ± 3.9	0.02
Age (years)	65.7 ± 7.6	58.8 ± 14.8	0.16
Diabetes	38.1%	20.6%	0.16
Smoking	22.7%	30.3%	0.54
Anemia	15.4%	30.9%	0.27
Immunosuppression	0	0	-

Values are reported as mean ± standard deviation or percentage frequency. p value was calculated as indicated in “Materials and methods” section

These analyses were conducted independent of the type of dressing used after the surgical procedure. The study identified an elevated BMI as the sole risk factor statistically associated with an increased likelihood of surgical site occurrence (*P* = 0.02). In contrast, advanced age (*P* = 0.16) and diabetes (*P* = 0.16), with a risk of 38.1% compared to 20.6% in non-diabetic patients, approached this threshold but did not achieve statistical significance. In contrast, in this study, smoking and anemia were not found to be associated with an increased risk of local complications; specifically, complications occurred in 22.7% of smokers compared to 30.3% of non-smokers (*P* = 0.54), and in patients with anemia, the risk was 15.4% compared to 30.9% in non-anemic patients (*P* = 0.27). Furthermore, none of the study participants were immunosuppressed.

For further evaluation, patients were divided into two groups: the control group (*n* = 34) and the group treated with sNPWT (PICO7®) (*n* = 21). Before recruitment, anthropometric and anamnestic data for each patient were collected, including age, body mass index, diabetes, smoking status, anemia, and immunosuppression. Parameters such as standard deviation, absolute frequency, and percentage frequency are shown in Table 6 for each item.

**Table 6:** Characteristics of the study groups

Risk factor	Control group (*n* = 34)	PICO 7 group (*n* = 21)
Body mass index (kg/m^2^)	28.9 ± 5.0	27.3 ± 4.3
Age (years)	59.7 ± 13.4	61 ± 14.6
Diabetes	13 (38.2%)	8 (38.1%)
Smoking	12 (35.3%)	12 (47.6%)
Anemia	6 (17.6%)	7 (33.3%)
Immunosuppression	0	0

Values are reported as mean ± standard deviation or absolute and percentage frequency

Statistical evaluation demonstrated that the control and PICO7® groups were homogeneous for each analyzed item; therefore, these two groups were comparable. This means that differences in the primary endpoint (surgical site occurrences, especially dehiscence) or secondary endpoint (need for surgical reoperation) are only correlated with the use or non-use of sNPWT.

## Discussion

Preventing surgical site occurrences (SSOs) in incisional hernias repaired with prosthetic mesh is crucial to ensure optimal patient outcomes. SSOs, such as infections or wound dehiscence, can compromise mesh integrity, increase the risk of hernia recurrence, and lead to significant morbidity. Effective prevention strategies are essential for minimizing complications, reducing healthcare costs, and improving long-term surgical success.

Our retrospective study revealed that Body Mass Index (BMI) was the only significant predictive factor for wound complications in clean-contaminated abdominal wall surgery (*P* = 0.02). This finding is consistent with existing literature, which has extensively documented the adverse effects of elevated BMI on wound healing. The mechanisms underlying this relationship are multi-faceted. An increased BMI leads to greater tension at the surgical site, potentially compromising wound closure and stability. In addition, excess adipose tissue can impair vascular perfusion, limiting the delivery of oxygen and nutrients that are essential for healing. Furthermore, obesity is associated with a chronic pro-inflammatory state, which can interfere with normal fibroblast function and collagen deposition, both crucial components of the wound healing process [24, 25].

This aligns with findings in the literature, such as studies emphasizing that high BMI correlates with an increased likelihood of delayed healing and infection following abdominal surgeries [26, 27].

Although age and diabetes also appeared to correlate with higher SSO rates, these associations did not reach statistical significance in this study.

Interestingly, this study did not find statistically significant associations between SSOs and other commonly cited risk factors, such as smoking and anemia. This divergence from previous findings highlights the complex nature of SSOs and the potential influence of the study design and cohort characteristics on research outcomes. As noted by Jones et al. [26], the impact of factors such as patient comorbidities, smoking, and diabetes on wound healing can vary depending on the specific study context and patient population. These inconsistencies across studies emphasizes the need for standardized research methodologies and reporting practices in the field of surgical site infections. By adopting more uniform approaches, researchers can better reconcile discrepancies between findings, improve the quality and comparability of results, and enhance our understanding of SSO risk factors and prevention strategies.

The rationale for the use of negative pressure wound therapy (NPWT) in surgical wounds is primarily based on its ability to reduce wound-related complications, such as infection, dehiscence, and seroma formation. In the context of closed laparotomy incisions, NPWT has been studied for its benefits in managing high-risk surgical wounds, particularly in general and colorectal surgeries [28, 29].

Bueno-Lledò et al. [30] already reported a significant reduction in the incidence of surgical site infections at 30 days postoperatively and significantly reduces postoperative length of stay in median laparotomy when sNPWT is used.

While the current study did not yield statistically significant differences between the PICO7® group and the control group, the observed trends were encouraging. The lower incidence of surgical site occurrences (SSOs) in the sNPWT group (19.05% vs. 32.35%) and fewer reoperations (10.53% vs. 17.65%) suggest a potential benefit of this dressing. These findings align with those of previous research, such as the study by Hopkins et al. [31] which reported reductions in SSOs and other wound complications with sNPWT dressings, albeit using a different device. The potential advantages of sNPWT are particularly pronounced in high-risk patients, such as those with an elevated BMI, diabetes, or advanced age.

In this study, *the need for surgical reintervention* referred to a secondary operation required because of a local complication confined to the surgical wound. Our findings suggest that in our experience, the use of PICO7® reduces this outcome.

However, it is crucial to note the limitations of sNPWT in addressing deep abdominal complications. The study emphasizes that while Pico7 shows promise in managing superficial wound issues, it does not significantly impact the incidence of surgical reintervention necessitated by deep abdominal complications. This distinction is important for healthcare providers when determining the appropriate use of sNPWT devices such as PICO7® in postoperative care. This suggests that while sNPWT may be beneficial for certain aspects of wound management, a comprehensive approach to patient care is still necessary, particularly for patients at risk of or experiencing deep abdominal complications.

The preliminary findings from the obese cohort, although not statistically significant, offer promising insights into the potential benefits of using PICO7® for postoperative surgical site management. The observed trend suggests that implementing the PICO7® system may contribute to a reduction in surgical site occurrences (SSOs) among obese patients, a population known to be at a higher risk of such complications.

Larger studies with larger sample sizes are needed to confirm these preliminary results and to better understand their broader clinical implications.

Despite the promising results, the limitations of this study must be acknowledged. The retrospective design, relatively small sample size, and single-center setting may have affected the generalizability of the results. Variability in xiphopubic distance among patients introduces potential heterogeneity in the surgical approach and outcomes. Furthermore, the assessment of local complications, particularly the need for reintervention, relies heavily on the subjective judgment of the surgical team, which could introduce bias. Future studies with larger multicenter cohorts and standardized evaluation criteria are needed to confirm and expand upon these findings. Larger multicenter randomized trials are warranted to confirm these findings and clarify the optimal patient selection criteria for sNPWT use. Further research could also explore long-term outcomes, as sNPWT might have an impact on late complications or recurrences that were not captured in this study’s 30-day follow-up period.

## Conclusions

This study suggests that single-use negative pressure wound therapy (sNPWT) with PICO7® dressing could offer a protective effect against surgical site occurrences and the need for surgical reoperation in patients undergoing open incisional hernia repair with mesh, although statistical significance was not achieved. These findings support the role of sNPWT as a potentially valuable tool in managing high-risk patients, particularly those with an elevated BMI. While our results indicate a trend towards reduced SSOs and reoperations, further high-quality studies are required to establish stronger evidence and identify the patient profiles most likely to benefit from this intervention.

## Data Availability

No datasets were generated or analysed during the current study.
